# Introducing a special collection of CME articles about long-acting injectable antipsychotics

**DOI:** 10.1017/S1092852925100722

**Published:** 2025-12-24

**Authors:** Leslie Citrome

**Affiliations:** Department of Psychiatry and Behavioral Sciences, New York Medical College, Valhalla, USA

## Abstract

Long-acting injectable (LAI) antipsychotics are not routinely offered and, thus, are underutilized despite their many advantages over oral formulations. In this special collection of articles, the reader will find overviews of the art and science of prescribing this important treatment option. Guidance is offered regarding incorporating LAIs in treatment planning, including inpatient, outpatient, and jail settings. Reviewed is the evidence surrounding the use of LAIs for patients in their first episode of schizophrenia, as well as switching from oral agents and other common issues that come up in day-to-day practice. Also provided is a comprehensive summary of each of the currently available formulations of LAIs, and some pragmatic reasons why one would be considered over another. In the end, the reader will come away with the notion that LAIs are not a “last resort” but an important and useful treatment modality that ought to be considered more often.

Imagine the availability of safe and effective long-acting injectable (LAI) preparations of antihypertensive medications.^1^ This formulation would be met with great fanfare and would make a major impact on the health of millions of people. Based on convenience, this modality could be preferred by many, especially with longer intervals between injections. In view of the high rates of partial or nonadherence with oral antihypertensive medication, including with yours truly,^2^ ^,^^3^ an LAI of an antihypertensive medication would equate to guaranteed delivery of a life-saving medication. It would be highly valued, offered early, and with few obstacles in its dissemination.

Although the revised American Psychiatric Association guidelines for the care of people with schizophrenia calls out that LAI antipsychotics should be considered in instances of poor or uncertain adherence, as well for patients who would prefer this modality,^4^ this antipsychotic medication formulation is not universally highly valued, is generally not offered early, and is fraught with obstacles. That is unfortunate. We can do better.

In this special collection of articles regarding LAI antipsychotics, the reader will find overviews of the art and science of prescribing this important treatment option. Weiden^5^ starts us off with a commentary “Lies and LAIs,” where the argument is made that LAI antipsychotics provide the information necessary to help clarify treatment response, avoid adherence blaming/shaming, enhance the therapeutic alliance, and facilitate patient-centered care. The figures and tables will be useful for those creating teaching materials.

Next is a guide to incorporating LAI antipsychotics in treatment planning, including inpatient, outpatient, and jail settings.^6^ Citrome and Matthews leveraged their clinical experience to place the medical literature into perspective and offered various tips.

LAI antipsychotics are not always considered for patients in their first episode of schizophrenia. Correll^7^ made a compelling case that LAI antipsychotics can be preferable to oral medications in this population. Exhaustively referenced, the pros and cons are reviewed in a clinically relevant manner that can be applied to thoughtful shared decision-making with patients and their families.

Guidance on starting LAI antipsychotics, including switching from oral agents, is offered by Grady and Cutler.^8^ Nuances regarding maintenance treatment is also discussed.

Saklad^9^ systematically reviewed common issues that come up in the day-to-day use of LAI antipsychotics. These include storage, reconstitution, injection site selection, injection technique, initiation regimens, missed doses, insufficient efficacy, drug–drug interactions, recreational drug use, common adverse effects, and addressing the return to oral formulations.

Finally, Citrome^10^ went over each of the currently available formulations of LAI antipsychotics, and some pragmatic reasons why one would be considered over another. Evidence for both acute and maintenance treatment is reviewed using the metrics of number needed to treat and number needed to harm.^11^

Each article can stand alone, and there are no prerequisites for reading each one. All of the authors are enthusiastic about this valuable treatment option, and references are thorough. Read one article or read them all! You’ll be glad you did.
